# An orb-weaver spider exploits an ant–acacia mutualism for enemy-free space

**DOI:** 10.1002/ece3.930

**Published:** 2014-01-02

**Authors:** John D Styrsky

**Affiliations:** Department of Biology, Lynchburg CollegeLynchburg, Virginia, 24503

**Keywords:** Acacia ant, ant-acacia, enemy-free space, exploitation, mutualism, orb-weaver spider, Panama

## Abstract

Exploiters of protection mutualisms are assumed to represent an important threat for the stability of those mutualisms, but empirical evidence for the commonness or relevance of exploiters is limited. Here, I describe results from a manipulative study showing that an orb-weaver spider, *Eustala oblonga*, inhabits an ant-acacia for protection from predators. This spider is unique in the orb-weaver family in that it associates closely with both a specific host plant and ants. I tested the protective effect of acacia ants on *E. oblonga* by comparing spider abundance over time on acacias with ants and on acacias from which entire ant colonies were experimentally removed. Both juvenile and adult spider abundance significantly decreased over time on acacias without ants. Concomitantly, the combined abundance of potential spider predators increased over time on acacias without ants. These results suggest that ant protection of the ant-acacia *Acacia melanocerus* also protects the spiders, thus supporting the hypothesis that *E. oblonga* exploits the ant–acacia mutualism for enemy-free space. Although *E. oblonga* takes advantage of the protection services of ants, it likely exacts little to no cost and should not threaten the stability of the ant–acacia mutualism. Indeed, the potential threat of exploiter species to protection mutualisms in general may be limited to species that exploit the material rewards traded in such mutualisms rather than the protection services.

## Introduction

Mutualism is a reciprocal interaction in which each of two species consumes a resource that the other provides (Holland et al. 2005; Ferrière et al. 2007; Holland and DeAngelis 2010). These resources are typically either material rewards (e.g., nutrients, shelter) or services (e.g., dispersal, defense) and can be thought of as commodities traded between species to provide net benefits to both (Janzen 1985; Schwartz and Hoeksema 1998).

The availability of traded commodities, however, also attracts exploiters, defined broadly as individuals of a species outside a mutualism that obtain a benefit offered to a mutualist but do not reciprocate (Bronstein 2001; Yu 2001; Ferrière et al. 2002). Such third-party exploitation is taxonomically and ecologically pervasive (Mainero and Martinez del Rio 1985; Bronstein 2001; Ferrière et al. 2007). Both rewards and services are commonly exploited, although the number of reported cases seems to vary depending on the type of mutualism. In transportation mutualisms (nutritional rewards traded for dispersal of gametes or offspring), exploitation of both rewards and dispersal services has been widely documented (Bronstein 2001). Familiar examples include birds that have evolved to rob nectar without pollinating flowers (Inouye 1983) and plants that have evolved nectarless flowers that attract pollinators but provide no reward (Gilbert et al. 1991).

In protection mutualisms (nutritional rewards traded for defense), rewards are also commonly exploited (Bronstein 2001). Certain ant species, for example, collect rewards provided by plants to attract mutualist plant-defending ants, but fail to protect the plants from herbivores or competitors (Raine et al. 2004; Clement et al. 2008). Despite numerous anecdotal reports, however, there are notably fewer empirical examples of third-party exploitation of the services offered in protection mutualisms. Building nests in ant-defended acacias, for instance, has long been thought to provide certain Neotropical birds indirect defense against nest predators (e.g., Skutch 1945; Janzen 1969; Young et al. 1990), but to date only one study has tested this hypothesis experimentally, finding limited support for it (Oliveras de Ita and Rojas-Soto 2006). A few other cases of exploitation of protection services are apparently fortuitous. A coccinellid beetle, for example, has evolved to infiltrate an ant–scale mutualism to gain unimpeded access to its scale prey, but gains the added bonus of indirect protection from parasitoid wasps by the scale-tending ants (Liere and Perfecto 2008).

Here, I document a novel example of exploitation of defense services involving an unusual interaction between an orb-weaver spider and an ant–acacia mutualism. The Neotropical orb-weaver spider *E. oblonga* constructs its webs among the branches of the ant-acacia *Acacia melanocerus*. The spiders occupy their webs at night, but rest during the day on the plant surface (stems, thorns, and leaves) in the midst of vigorously patrolling workers of the acacia&s obligate ant partner *Pseudomyrmex satanicus*. The spiders are behaviorally adapted to avoid being attacked by the aggressive, plant-defending ants (Garcia and Styrsky 2013), but if they are discovered and caught, the ants can kill them. Myrmecophily (living in close association with ants) is unusual in spiders (Cushing 1997, 2012), but *E. oblonga* (and a sister species, *Eustala illicita*) are unique in that they are the only known myrmecophilous spiders in the large and diverse orb-weaver family (Araneidae; Garcia and Styrsky 2013). As an orb-weaver, *E. oblonga* does not feed on the rewards provided by the plant or on the acacia ants, but rather on flying insects the spiders capture passively in their webs (J. D. Styrsky, unpubl. data). I hypothesized, therefore, that *E. oblonga* inhabits *A. melanocerus* to exploit the plant-protection services provided by the ants for defense against their own natural enemies (i.e., enemy-free space (Jeffries and Lawton 1984)). I tested this hypothesis by removing whole colonies of acacia ants from a group of acacias and comparing spider abundance over time on acacias with and without defending ants. I predicted that there would be fewer spiders over time and a concomitant increase in potential spider predators on acacias from which ants had been removed relative to acacias with intact ant colonies.

## Materials and Methods

This study was conducted in July and August 2009 in a 100-ha study area in the Rio Limbo basin of Parque Nacional Soberanía in central Panama (9°9′35″N, 79°44′36′′W). The habitat in the study area is characterized as lowland tropical moist forest and ranges in age from 40 to 400 years old (Robinson et al. 2000b). The ant-acacia *A. melanocerus* is endemic to Panama and occurs in low densities in a limited area of the Atlantic side of the former Canal Zone (Janzen 1974). Like other swollen-thorn acacias, *A. melanocerus* provides nutritional rewards (extrafloral nectar and Beltian bodies) and domatia (hollow thorns) to an obligate mutualist ant species in exchange for ant defense from herbivores and competing plants (Janzen 1966). The ant mutualist *P. satanicus* aggressively patrols its host plants 24 h a day, and the workers rapidly attack and remove any other arthropods they encounter (Janzen 1966). Despite the ants& effectiveness as plant bodyguards, however, the orb-weaver spider *E. oblonga* occurs in abundance on adult acacias, where there can be hundreds of individuals (adults and juveniles) present (J. D. Styrsky, pers. observ.).

**Figure 1:**
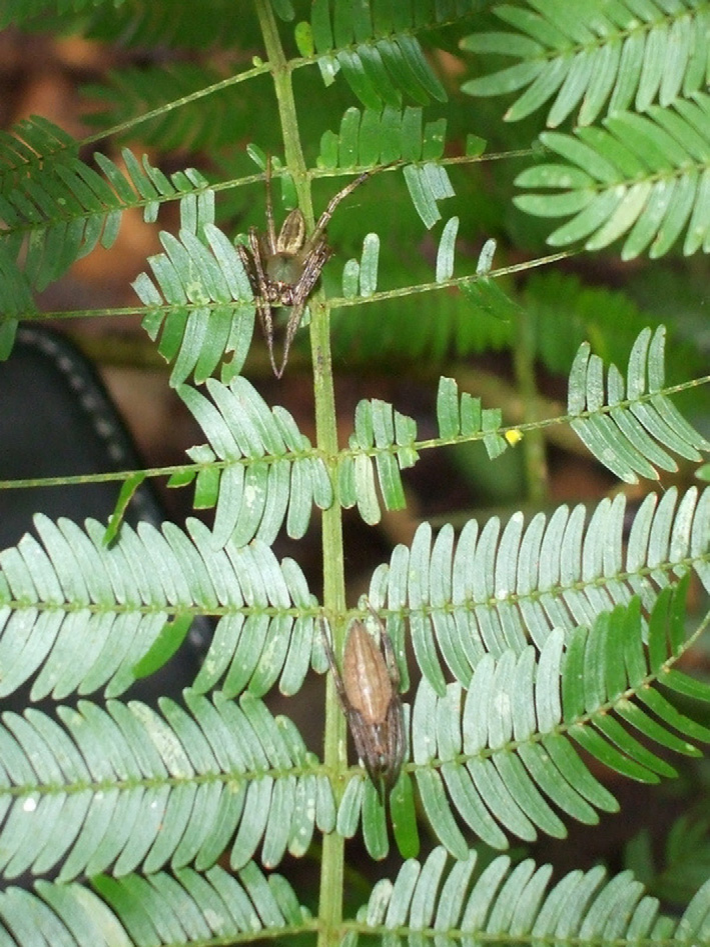
Adult male (top) and adult female (bottom) *Eustala oblonga* crouching on a compound leaf of the ant-acacia *Acacia melanocerus* in Parque Nacional Soberanía, central Panama. Note the male&s considerably longer legs, larger prosoma, and smaller opisthosoma. The female&s opisthosoma is distended because she is gravid.

*Eustala oblonga* is also endemic to Panama (Platnick 2012), but very little is known about its distribution or natural history. Adults range in body length from about 7 to 11 mm with females averaging larger than males (see Garcia and Styrsky 2013 for additional details). Adult males and females are sometimes found together as pairs (Fig. 1), but their courtship and mating behavior has not been described. Females construct egg sacs on the surface of larger branches and remain near them until the eggs hatch. Hatchling spiders cluster in diffuse webbing near the egg sac and then move to acacia leaflets where they construct small orb webs at night. Juvenile spiders darken as they age, changing from a light yellow green color that matches the undersides of the leaflets to a darker gray brown color that matches the color of the acacia bark. The spiders are behaviorally adapted to avoid ant aggression by remaining motionless while resting on the plant surface and not reacting to patrolling workers (Garcia and Styrsky 2013).

Curiously, *E. oblonga* is found only rarely on other understory plants. In July 2008, I surveyed 50 randomly chosen sapling acacias and another 50 randomly chosen, neighboring (within a 5-m radius) plants of similar size and structure. *E. oblonga* abundance ranged from 0 to 74 spiders (mean ± standard deviation (SD) = 13.4 ± 17) on acacias (spiders occurred on 43 of 50 acacias), whereas only one individual was found on only one of the 50 neighboring nonacacias. *E. illicita*, a sister species of *E. oblonga,* has a similarly close association with another species of ant-acacia, *Acacia collinsii*, on the Pacific side of the Canal Zone (Hesselberg and Triana 2010). This high level of host–plant specificity is remarkable, because it is uncharacteristic of the orb-weaver spider family and has not been documented previously.

I tested the hypothesis that *E. oblonga* exploits the ant–acacia mutualism for enemy-free space in a field experiment in which I randomly assigned 30 sapling acacias (1.5–2.5 m tall) to one of two treatments: ant removal (*n *=* *15) or control (*n *=* *15). Prior to the experiment, I attempted to remove patrolling acacia ants on three separate acacias using an aspirator. This technique was not sufficient, however, because I could not remove the nursery maids or the developing brood from within the hollow thorns, and patrolling workers soon reappeared on the acacias. Therefore, I removed whole acacia ant colonies from experimental acacias using an aspirator to collect patrolling workers first and then injecting 0.1–0.2 cc of dilute insecticide (0.05= cypermethrin [Cyper WP; Control Solutions, Inc., Pasadena, TX]) into every hollow thorn. The dilute insecticide was delivered using a 23-gauge butterfly needle attached to a 3-cc syringe with a 30-cm length of flexible rubber tubing. The needle was threaded through the opening in the tips of the thorns to limit mechanical disturbance to the plant. This procedure killed any adult ants inside the thorns and all the ant eggs, larvae, and pupae. Great care was taken to prevent any insecticide from getting on the surface of the acacias, but if any residual amount of this highly water-soluble insecticide remained, it should have been washed off by the frequent wet season precipitation. Letourneau and Dyer (1998) and Dyer and Letourneau (1999) used the same technique to manipulate ant presence and absence on *Piper* plants in Costa Rica without affecting other arthropods on the plants.

I recorded the number of all juvenile and adult *E. oblonga* on the experimental and unmanipulated control acacias 3–4 days before and 3–4 days after application of the insecticide, and then once a week for the next 5 weeks. At these same intervals, I also recorded the number of acacia ants (to confirm the experimental treatment) on three haphazardly chosen branches and the number and identity (order, family, or species) of all other macroscopic invertebrate and vertebrate animals on the entire plant. Any stray acacia ants found on the experimental acacias during the weekly censuses were removed.

I tested for effects of ant presence and absence on the abundance of juvenile and adult *E. oblonga* separately, and on the abundance of all potential spider predators and all potential spider prey using repeated-measures analysis of variance (ANOVA; SAS proc mixed; SAS Institute Inc 2001). I included the number of leaves on each acacia as a covariate to control for effects of acacia size. Data were log-transformed to meet statistical assumptions. Post hoc pairwise comparisons of least squares means were used to test for differences between experimental and control acacias in specific weeks. Experiment-wise error rate in all post hoc tests was controlled using sequential Bonferroni corrections.

## Results

**Figure 2:**
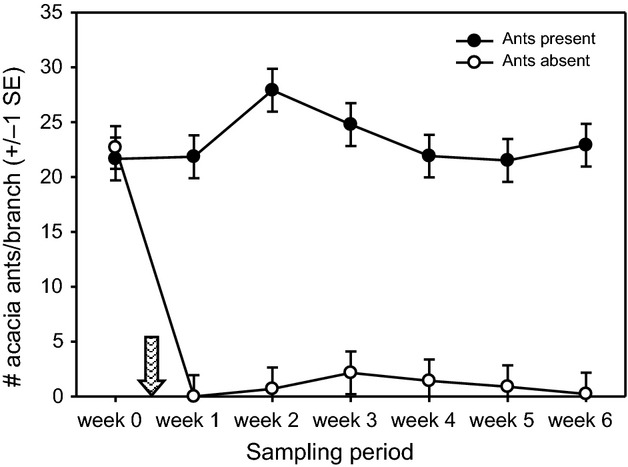
Mean acacia ant abundance (number of ants averaged over three branches) over time on experimental (ants killed with dilute insecticide) and control acacias (unmanipulated). The ant-removal treatment was applied between week 0 and week 1 (shown by the hatched arrow).

The application of the dilute insecticide successfully removed acacia ants from the experimental acacias (treatment × time *F*_6,168_ = 29.58, *P *<* *0.0001). The abundance of patrolling workers on branches was statistically indistinguishable from zero on experimental acacias after application of the treatment, but remained high on control acacias over the entire sampling period (mean ± 1 standard error (SE) = 23.3 ± 1.3 ants per branch; Fig. 2).

**Figure 3:**
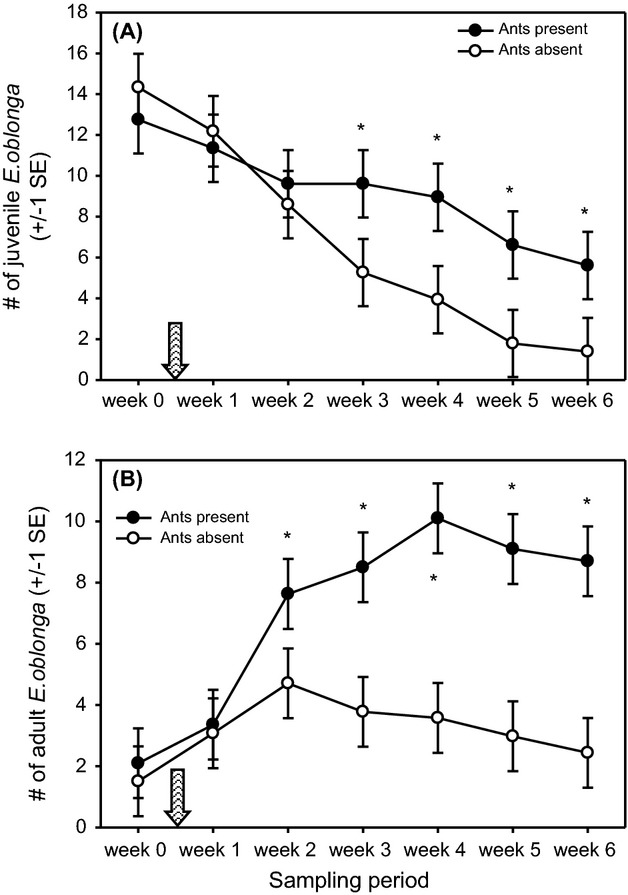
Mean abundance of (A) juvenile and (B) adult *Eustala oblonga* (mean number of spiders per acacia) over time on experimental (ants absent) and control acacias (ants present). The ant-removal treatment was applied between week 0 and week 1 (shown by the hatched arrow). Asterisks indicate significant differences between the two treatments during specific weeks.

The experimental removal of acacia ants did not affect *E. oblonga* immediately, but effects were apparent over time. Juvenile spider abundance decreased over the sampling period on both experimental and control acacias (Fig. 3A), reflecting a seasonal change in demography as juvenile spiders matured into adults (time effect: *F*_6,168_ = 24.37, *P *<* *0.0001). Juvenile spider abundance decreased significantly more over time, however, on acacias without ants (treatment × time effect: *F*_6,168_ = 29.58, *P *<* *0.0001). Consequently, juvenile spider abundance was significantly lower on acacias without ants during each of the last 4 weeks of the sampling period (Fig. 3A).

In contrast, adult spider abundance initially increased on both experimental and control acacias, again reflecting a seasonal demographic shift as juveniles matured into adults (Fig. 3B). Adult spider abundance increased more slowly on acacias without ants, however, and then decreased over the last 4 weeks of the sampling period (treatment × time effect: *F*_6,168_ = 6.83, *P *<* *0.0001). Consequently, adult spider abundance was significantly lower on acacias without ants during each of the last 5 weeks of the sampling period (Fig. 3B).

**Figure 4:**
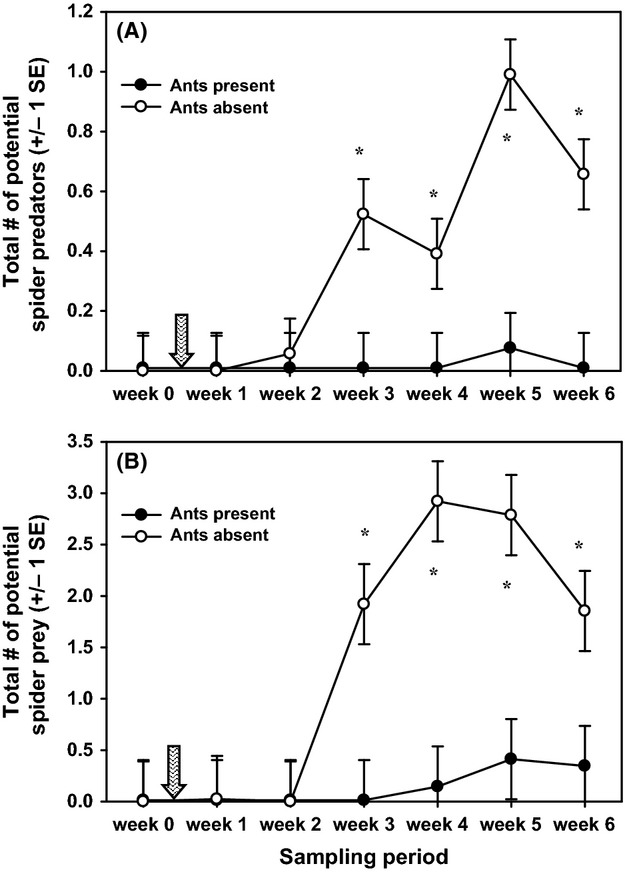
Mean abundance of (A) potential spider predators (mean number of predators per acacia) and (B) potential spider prey (mean number of prey per acacia) over time on experimental (ants absent) and control acacias (ants present). The ant-removal treatment was applied between week 0 and week 1 (shown by the hatched arrow). Asterisks indicate significant differences between the two treatments during specific weeks.

I never directly observed a predator consuming any of the spiders, but I categorized other animals recorded on the acacias as potential spider predators based on their general diet (i.e., if they are known to prey on arthropods, including spiders). Potential spider predators included four bird species (Song Wren [*Cyphorhinus phaeocephalus*], Spotted Antbird [*Hylophylax naevioides*], Black-crowned Antshrike [*Thamnophilus atrinucha*], and Blue-crowned Manakin [*Lepidothrix coronata*]), one lizard species (*Anolis limifrons*), jumping spiders (Salticidae), crab spiders (Thomisidae), assassin bugs (Reduviidae), predaceous stink bugs (Pentatomidae: Asopinae), predaceous beetles (Carabidae), praying mantises (Mantodea), one spider wasp (Pompilidae), and ants in the subfamily Ponerinae. Combining all observations, the abundance of potential spider predators significantly increased over the sampling period on acacias without ants, whereas few, if any, were recorded on control acacias (treatment × time effect: *F*_6,168_ = 5.56, *P *<* *0.0001). Consequently, the abundance of potential spider predators was significantly higher on acacias without ants than on control acacias during each of the last 4 weeks of the sampling period (Fig. 4A).

*Eustala oblonga* occupies its webs primarily at night, but to obtain some sense of whether the exclusion of acacia ants altered prey availability for the spiders, I compared the abundance of any flying insects observed on acacias with and without ants. A preliminary analysis of prey remains in webs in a previous study indicated that *E. oblonga* captures and feeds on several groups of Hemipterans (leafhoppers [Flatidae], froghoppers [Cercopidae], treehoppers [Membracidae], and unidentified Heteropterans), parasitoid wasps (Hymenoptera), and small moths (Lepidoptera; J. D. Styrsky, unpubl. data). In addition to these known groups of prey, I also considered as potential prey the following insects: damselflies (Odonata), roaches (Blattaria), katydids (Tettigoniidae), grasshoppers (Acrididae), tree crickets (Gryllidae), cicadas (Cicadidae), stilt bugs (Berytidae), stink bugs (Pentatomidae), assassin bugs (Reduviidae), Dipterans including crane flies (Tipulidae), stilt-legged flies (Micropezidae), and several unidentified fly taxa, predaceous beetles (Carabidae), scarab beetles (Scarabaeidae), leaf beetles (Chrysomelidae), weevils (Curculionidae), and an unidentified bee (Hymenoptera). Combining all observations, the abundance of potential spider prey increased significantly over the sampling period on acacias without ants, compared with control acacias (treatment × time effect: *F*_6,166_ = 7.54, *P *<* *0.0001). Consequently, the abundance of potential spider prey was significantly higher on acacias without ants than on control acacias during each of the last 4 weeks of the sampling period (Fig. 4B).

## Discussion

The natural history of *E. oblonga* is intriguing because unlike any other known orb-weaver spider (other than sister species *E. illicita*), this species shows a high degree of specificity for a particular host plant, which is also inhabited by aggressive, plant-defending ants. Further, *E. oblonga* is behaviorally adapted to avoid ant aggression and attack: a previous field experiment showed that the spiders apparently “hide” from acacia ants by refraining from movement in general while resting on the plant surface and specifically by not attempting to flee if they are confronted by patrolling ants (Garcia and Styrsky 2013). Here, I show that by living on the ant-acacia *A. melanocerus*,*E. oblonga* is likely protected from its own natural enemies. Both juvenile and adult spider abundance were significantly lower over time on acacias from which acacia ants were experimentally removed relative to control acacias with intact ant colonies. Concomitantly, the abundance of potential spider predators increased over time on acacias from which ants were removed. Spider predation was never actually observed, however, leaving open the possibility that the spiders voluntarily left the acacias in response to the ant-removal treatment. Other orb-weaver spiders relocate to new foraging sites in response to low prey availability or high rates of web damage (Nakata and Ushimaru 1999; Chmiel et al. 2000). *Eustala oblonga* does not prey on acacia ants, however, and the abundance of potential spider prey on acacias from which ants were removed actually increased. Further, abandoning the experimental acacias to find another acacia with an intact ant colony is seemingly risky because *A. melanocerus* is so sparsely distributed in the forest understory.

Of greater potential concern is that the dilute insecticide used to remove acacia ants could have detrimentally affected the spiders. This explanation is unlikely for three reasons. Firstly, exposure to cypermethrin results in rapid death, yet spider abundance on experimental acacias did not decrease immediately after applying the insecticide (see Fig. 3A and B). Secondly, the spiders could not come into physical contact with the insecticide anyway because they do not enter the hollow thorns. Even if a drop of dilute insecticide was left inadvertently on the plant surface, it would have been washed away by rain long before spider abundance began to decline on experimental plants 3 weeks after insecticide application (again, see Fig. 3A and B). Thirdly, when applied to plants, cypermethrin is not absorbed and does not become plant systemic (e.g., Gaughan and Casida 1978). Although I do not present the data here, the abundance of leaf-feeding caterpillars also increased significantly over time on acacias from which ants were removed using insecticide (J. D. Styrsky, unpubl. data), indicating that the insecticide was not transported throughout the plants& tissues. The results, therefore, provide correlative support for the hypothesis that *E. oblonga* exploits the plant-defense services traded in the ant–acacia mutualism for enemy-free space. Experimental removal of acacia ants resulted in decreased spider abundance (despite increased potential spider prey) and increased abundance of potential spider predators.

The jumping spider *Bagheera kiplingi* is also obligately associated with certain species of Neotropical ant-acacias, which it exploits by consuming the food rewards provided by the plants for their ant mutualists (Meehan et al. 2009). Whether inhabiting the ant-acacias also provides *B. kiplingi* indirect protection from predators is certainly likely but has not been tested. In fact, exploitation of defense services in protection mutualisms has not been widely documented in general, which is surprising given the ubiquity of two common types of these mutualisms: those between ants and ant-plants (Davidson and McKey 1993), and those between ants and honeydew-producing hemipteran insects (Styrsky and Eubanks 2007).

Several anecdotal reports and observational studies suggest many other organisms, including a cockroach species that glues its ootheca to acacia branches (Deans and Roth 2003) and several species of birds (e.g., Skutch 1960; Janzen 1969) and social wasps (DeJean et al. 2001) that construct their nests in acacias, exploit ant–acacia mutualisms for the protective services of acacia ants. Oliveras de Ita and Rojas-Soto (2006), however, provide the only empirical support for this hypothesis in any ant–plant mutualism and it is fairly weak. Using artificial nests containing plasticine eggs, these authors found that both daily survival probability and overall nest success were significantly greater for nests placed in the ant-acacia *Acacia hindsii* than in neighboring trees (nonacacias) without ants. Other, nonexperimental studies that compared the fate of nests constructed naturally in ant-acacias versus nonacacias have reported no difference in nest success between acacias and nonacacias (Robinson et al. 2000a) or even decreased nest success in ant-acacias (Young et al. 1990). Manipulating the presence and absence of acacia ants provides a stronger test of the hypothesis that other organisms inhabit ant-acacias to gain enemy-free space, but to date only the experiment reported here has employed this design.

Other organisms have been documented inhabiting ant-acacias, including a coreid bug (Reid 2010) and the shelter-building larvae of a gelechiid moth (Eubanks et al. 1997), but these species evidently have evolved the ability to evade acacia ants to consume leaf tissue rather than the nectar or food rewards provided by the acacias. Whether these herbivores are indirectly protected by their association with ant-acacias is often suspected or even assumed, but not tested. Further, exploitation of ant–hemipteran mutualisms has also been reported, but the exploiter species either themselves engage in protection mutualisms with ants, as in the case of a lycaenid butterfly that oviposits preferentially in the presence of an ant–treehopper association (Kaminski et al. 2010), or they are specialist predators or parasitoids that have evolved the ability to escape the notice of tending ants to access their hemipteran prey, but are also indirectly protected by tending ants (Völkl 1992; Liere and Perfecto 2008).

How mutualisms persist in the presence of third-party exploiters has attracted recent theoretical interest, leading to the development of several mathematical models that identify specific conditions under which exploited mutualisms are ecologically viable (e.g., Law et al. 2001; Ferrière et al. 2007; Jones et al. 2009). These models apply to dispersal mutualisms specifically and make two common assumptions: (1) mutualist and exploiter species compete for a traded commodity (generally the reward); and (2) exploitation imposes a cost on the host mutualist (i.e., the species providing the reward), particularly in highly specialized mutualisms that involve significant investments in rewards. In contrast, in protection mutualisms in which a species outside the mutualism exploits only the protection services provided by one of the mutualists (and does not consume either mutualist), the costs of exploitation may be negligible because the protection services are not a limiting resource and no rewards are lost to unintended consumers. In the ant–acacia mutualism, for example, the presence of an exploiter species such as *E. oblonga* does not impose a cost on the host acacia by consuming rewards and presumably does not diminish the plant-protection services provided by the patrolling acacia ants. Previous work has shown that *P. satanicus* typically ignores *E. oblonga* on *A. melanocerus* (Garcia and Styrsky 2013); thus, the spiders may be taking advantage of a service without increasing the cost of that service to either the ants or the acacias. In this sense, the exploitation of the ant–acacia mutualism by *E. oblonga* is commensalistic rather than parasitic and should not destabilize the mutualism. *Eustala oblonga* may even provide an additional layer of defense for their host acacias in which the spider might be considered a mutualist partner of the plant, but this has not been tested.

Models of third-party exploitation also predict under certain circumstances that invasion of mutualisms by exploiters should alter the coevolutionary trajectory of mutualists in such a way as to generate long-term stability while accommodating the exploiter (Ferrière et al. 2007; Jones et al. 2009). As discussed above, the presence of *E. oblonga* may not exert significant selective pressure on either *P. satanicus* or *A. melanocerus*; however, *Pseudomyrmex*–*Acacia* mutualisms have apparently driven evolutionary changes in the orb-weaver genus *Eustala*. Both *E. oblonga* and an almost identical sister species, *E*. *illicita*, have evolved a unique and specialized association with two different ant-defended acacias. Based on the results presented here, this association has evolved in response to a commodity traded in ant–acacia mutualisms – the protection services of aggressive ants.
